# Supplementary material for: Oxidative Stress and Antioxidant Response in Populations of the Czech Republic Exposed to Various Levels of Environmental Pollutants
Source: Int J Environ Res Public Health. 2022 Mar 18;19(6):3609. doi: 10.3390/ijerph19063609 (PMC8955578; doi:10.3390/ijerph19063609)

Supplementary Figure S1. A map of the Czech Republic with highlighted localities in which analyzed samples were collected.

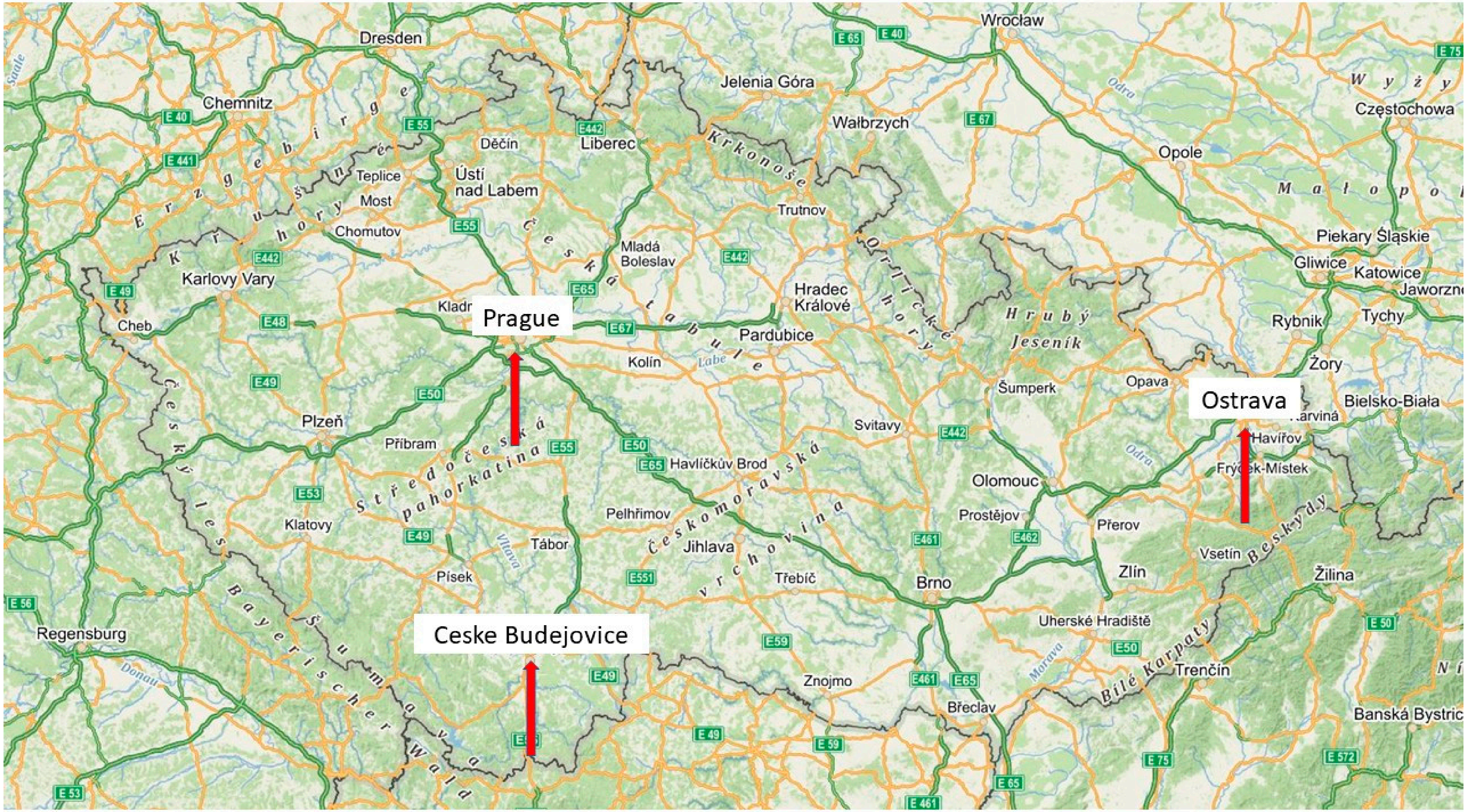

Supplement: Supplementary file 1 [file ijerph-19-03609-s001.zip › Supplementary Figure S1.pdf]
