# Supplementary material for: Oxidative Stress and Antioxidant Response in Populations of the Czech Republic Exposed to Various Levels of Environmental Pollutants
Source: Int J Environ Res Public Health. 2022 Mar 18;19(6):3609. doi: 10.3390/ijerph19063609 (PMC8955578; doi:10.3390/ijerph19063609)
Supplement: Supplementary file 1 [file ijerph-19-03609-s001.zip › Supplementary Tables S1 - S4-revised-clean.pdf]

Supplementary Table S1. Concentrations of plasma POPs and urinary PAH metabolites in samples collected in Ceske Budejovice, a rural locality

Variable	Ceske Budejovice (N=16) (Season 1) Mean ± SD	Median (min, max)	Ceske Budejovice (N=16) (Season 2) Mean ± SD	Median (min, max)	P
		**Polychlorinated biphenyls (ng/g lipid weight)**			
PCB 28	1.04 ± 1.00	0.85 (0.05, 4.30)	0.36 ± 0.24	0.31 (0.05, 0.83)	0.002
PCB 52	0.08 ± 0.05	0,05 (0.05, 0.20)	0.15 ± 0.22	0.05 (0.05, 0.77)	0.92
PCB 101	0.31 ± 0.26	0.38 (0.05, 0.78)	0.15 ± 0.15	0.05 (0.05, 0.42)	0.09
PCB 118	2.20 ± 1.11	1.97 (0.88, 4.37)	3.87 ± 3.21	3.39 (0.05, 12.1)	0.10
PCB 138	15.6 ± 7.39	15.0 (4.72, 28.9)	45.7 ± 22.8	41.1 (15.7, 92.5)	<0.001
PCB 153	36.2 ± 15.8	35.1 (15.3, 60.6)	74.7 ± 32.7	72.5 (27.7, 133.7)	0.001
PCB 170	17.8 ± 6.83	18.4 (7.40, 30.2)	27.3 ± 13.0	27.0 (8.17, 54.3)	0.02
PCB 180	43.6 ± 17.5	44.3 (20.7, 71.1)	72.1 ± 33.6	70.7 (16.3, 135.1)	0.01
		**Organochlorinated pesticides (ng/g lipid weight)**			
o,p'-DDE	0.07 ± 0.05	0.05 (0.05, 0.17)	0,05 ± 0.00	0.05 (0.05, 0.05)	0.04
p,p'-DDE	96.1 ± 61.5	76.1 (39.0, 280.4)	196.4 ± 151.7	154.0 (87.4, 708.2)	0.001
o,p'-DDD	0.05 ± 0.00	0.05 (0.05, 0.05)	0.05 ± 0.00	0,05 (0.05, 0.05)	1.00
p,p'-DDD	1.30 ± 1.83	0.66 (0.12, 7.63)	3.45 ± 6.21	1.68 (0.05, 25.91)	0.02
o,p'-DDT	0.31 ± 0.17	0.25 (0.25, 0.86)	0.45 ± 0.79	0.25 (0.25, 3.4)	0.60
p,p'-DDT	7.68 ± 14.1	3.48 (0.25, 57.9)	6.21 ± 21.2	0.25 (0.25, 85.4)	0.001
HCB	14.4 ± 6.40	12.5 (7.64, 29.1)	18.8 ± 8.76	15.8 (8.77, 37.0)	0.09
α-HCH	0.13 ± 0.21	0.05 (0.05, 0.75)	0.05 ± 0.00	0.05 (0.05, 0.05)	0.07
β-HCH	2.58 ± 2.85	2.44 (0.05, 11.5)	2.01 ± 6.26	0.05 (0.05, 24.6)	0.01
γ-HCH	0.06 ± 0.04	0.05 (0.05, 0.20)	0.43 ± 1.10	0.05 (0.05, 4.39)	0.25
		**Brominated flame retardants (ng/g lipid weight)**			
BDE 47	2.90 ± 4.96	1.02 (0.05, 17.9)	1.95 ± 0.55	1.94 (1.19, 3.01)	0.004
BDE 99	0.15 ± 0.00	0.15 (0.15, 0.15)	0.24 ± 0.29	0.15 (0.15. 1.28)	0.15
BDE 100	0.15 ± 0.00	0.15 (0.15, 0.15)	0.17 ± 0.09	0.15 (0.15, 0.51)	0.32
BDE 153	1.03 ± 3.54	0.15 (0.15, 14.3)	1.20 ± 3.98	0.15 (0.15, 16.1)	0.31
BDE 154	0.15 ± 0.00	0.15 (0.15, 0.15)	0.15 ± 0.00	0.15 (0.15, 0.15)	1.00

BDE 183	0.75 ± 2.40	0.15 (0.15, 9.74)	0.74 ± 2.36	0.15 (0.15, 9.58)	0.96
BDE 209	1.82 ± 2.23	0.75 (0.75, 8.65)	4.09 ± 13.4	0.75 (0.75, 54.2)	0.10
**Per- and polyfluoroalkylated substances (ng/mL plasma)**					
PFBS	0.01 ± 0.00	0.01 (0.01, 0.01)	0.01 ± 0.00	0.01 (0.01, 0.02)	0.04
PFHxS	0.34 ± 0.18	0.41 (0.07, 0.66)	0.35 ± 0.18	0.41 (0.08, 0.68)	0.90
PFOS	3.49 ± 1.82	3.94 (0.46, 6.32)	3.45 ± 1.68	4.2 (0.53, 5.44)	0.97
PFDS	0.01 ± 0.00	0.01 (0.01, 0.01)	0.01 ± 0.00	0.01 (0.01, 0.02)	0.32
PFBA	0.02 ± 0.01	0.02 (0.01, 0.05)	0.03 ± 0.01	0.02 (0.01, 0.05)	0.56
PFHpA	0.02 ± 0.02	0.01 (0.01, 0.07)	0.02 ± 0.02	0.01 (0.01, 0.08)	0.85
PFOA	1.05 ± 0.61	1.02 (0.09, 2.59)	1.18 ± 0.64	1.21 (0.14, 2.90)	0.41
PFNA	0.55 ± 0.29	0.62 (0.06, 1.01)	0.60 ± 0.30	0.65 (0.07, 1.09)	0.62
PFDA	0.26 ± 0.14	0.25 (0.06, 0.55)	0.37 ± 0.19	0.34 (0.07, 0.72)	0.12
PFUdA	0.08 ± 0.04	0.09 (0.03, 0.15)	0.12 ± 0.07	0.12 (0.04, 0.31)	0.06
PFDoA	0.02 ± 0.02	0.02 (0.01, 0.09)	0.03 ± 0.02	0.02 (0.01, 0.07)	0.97
PFTrDA	0.01 ± 0.01	0.01 (0.01, 0.03)	0.01 ± 0.01	0.01 (0.01, 0.05)	0.71
PFTeDA	0.01 ± 0.01	0.01 (0.01, 0.02)	0.01 ± 0.00	0.01 (0.01, 0.02)	0.38
**Monohydroxylated PAH metabolites (µg/g creatinine)**					
1-OH-NAP	0.59 ± 0.58	0.38 (0.04, 1.98)	1.23 ± 2.81	0.42 (0.07, 11.7)	0.73
2-OH-NAP	4.68 ± 3.34	3.22 (1.39, 13.3)	5.38 ± 5.87	3.21 (1.31, 24.6)	0.82
2-OH-FLUO	0.44 ± 0.32	0.38 (0.01, 1.25)	0.35 ± 0.33	0.29 (0.09, 1.42)	0.26
1-OH-PHEN	0.20 ± 0.19	0.13 (0.07, 0.80)	0.26 ± 0.26	0.18 (0.04, 1.02)	0.41
2-OH-PHEN	0.08 ± 0.10	0.06 (0.03, 0.44)	0.12 ± 0.08	0.09 (0.04, 0.33)	0.03
3-OH-PHEN	0.10 ± 0.07	0.08 (0.03, 0.27)	0.11 ± 0.10	0.08 (0.02, 0.35)	0.90
4-OH-PHEN	0.02 ± 0.03	0.02 (0.00, 0.09)	0.06 ± 0.08	0.02 (0.01, 0.28)	0.68
9-OH-PHEN	0.06 ± 0.03	0.06 (0.03, 0.11)	0.20 ± 0.33	0.08 (0.03, 1.29)	0.25
1-OH–pyrene	0.08 ± 0.06	0.06 (0.01, 0.20)	0.10 ± 0.06	0.10 (0.01, 0.23)	0.39

Supplementary Table S2. Concentrations of plasma POPs and urinary PAH metabolites in samples collected in Prague, a metropolitan locality

	Prague (N=56) (Season 1)		Prague (N=56) (Season 2)		
**Variable**	Mean ± SD	Median (min, max)	Mean ± SD	Median (min, max)	P
		**Polychlorinated biphenyls (ng/g lipid weight)**			
PCB 28	1.02 ± 1.23	0.54 (0.05, 4.33)	0.60 ± 1.02	0.31 (0.05, 7.00)	0.10
PCB 52	0.06 ± 0.04	0.05 (0.05, 0.29)	0.34 ± 0.53	0.05 (0.05, 3.31)	<0.001
PCB 101	0.31 ± 0.43	0.05 (0.05, 2.39)	0.40 ± 0.73	0.05 (0.05, 3.26)	0.66
PCB 118	2.08 ± 1.57	2.02 (0.05, 8.09)	4.28 ± 2.55	3.48 (1.19, 11.9)	<0.001
PCB 138	19.7 ± 15.6	16.7 (3.47, 78.3)	52.2 ± 41.1	39.9 (4.58, 224.6)	<0.001
PCB 153	43.3 ± 34.6	35.3 (5.99, 191.6)	85.6 ± 69.5	63.0 (10.2, 398.6)	<0.001
PCB 170	24.3 ± 18.8	19.9 (3.27, 98.1)	34.4 ± 27.4	27.5 (4.10, 148.3)	0.01
PCB 180	58.6 ± 44.5	47.1 (6.86, 232.3)	96.4 ± 77.7	82.9 (9.29, 396.3)	0.001
		**Organochlorinated pesticides (ng/g lipid weight)**			
o,p'-DDE	0.23 ± 0.30	0.15 (0.05, 2.00)	0.07 ± 0.11	0.05 (0.05, 0.65)	<0.001
p,p'-DDE	81.9 ± 87.8	55.4 (11.8, 574.2)	180.6 ± 172.2	124.5 (24.7, 997.4)	<0.001
o,p'-DDD	0.09 ± 0.11	0.05 (0.05, 0.67)	0.09 ± 0.13	0.05 (0.05, 0.65)	0.30
p,p'-DDD	0.91 ± 0.91	0.69 (0.05, 6.41)	1.61 ± 3.07	1.09 (0.05, 22.8)	0.05
o,p'-DDT	0.27 ± 0.13	0.25 (0.25, 1.23)	0.27 ± 0.15	0.25 (0.25, 1.33)	0.97
p,p'-DDT	3.11 ± 7.70	0.25 (0.25, 56.9)	3.81 ± 12.22	0.25 (0.25, 88.8)	0.61
HCB	12.1 ± 7.52	9.22 (2.28, 35.8)	19.1 ± 13.1	15.2 (4.54, 68.7)	<0.001
α-HCH	0.09 ± 0.31	0.05 (0.05, 2.35)	0.05 ± 0.00	0.05 (0.05, 0.05)	0.33
β-HCH	2.74 ± 2.74	1.89 (0.05, 13.5)	4.18 ± 6.65	0.05 (0.05, 28.5)	0.17
γ-HCH	0.17 ± 0.31	0.05 (0.05, 2.11)	0.65 ± 1.58	0.05 (0.05, 7.06)	0.34
		**Brominated flame retardants (ng/g lipid weight)**			
BDE 47	1.01 ± 0.98	0.74 (0.05, 3.87)	1.93 ± 0.87	1.81 (0.05, 5.08)	<0.001
BDE 99	0.24 ± 0.52	0.15 (0.15, 3.96)	0.29 ± 0.50	0.15 (0.15, 3.53)	0.07
BDE 100	0.17 ± 0.12	0.15 (0.15, 1.03)	0.18 ± 0.15	0.15 (0.15, 1.14)	0.54
BDE 153	0.16 ± 0.11	0.15 (0.15, 0.96)	0.26 ± 0.25	0.15 (0.15, 1.18)	0.002
BDE 154	0,15 ± 0.00	0.15 (0.15, 0.15)	0.16 ± 0.07	0.15 (0.15, 0.65)	0.31

BDE 183	0.17 ± 0.17	0.15 (0.15, 0.15)	0.18 ± 0.19	0.15 (0.15, 1.50)	0.54
BDE 209	5.65 ± 33.5	0.75 (0.75, 251.8)	35.0 ± 118.5	0.75 (0.75, 618.2)	0.73
**Per- and polyfluoroalkylated substances (ng/mL plasma)**					
PFBS	0.01 ± 0.01	0.01 (0.01, 0.11)	0.01 ± 0.01	0.01 (0.01, 0.04)	0.004
PFHxS	0.39 ± 0.18	0.40 (0.04, 0.74)	0.41 ± 0.19	0.43 (0.05, 0.80)	0.63
PFOS	3.23 ± 2.06	2.68 (0.26, 9.99)	3.46 ± 1.81	3.35 (0.24, 7.20)	0.34
PFDS	0.01 ± 0.00	0.01 (0.01, 0.01)	0.01 ± 0.00	0.01 (0.01, 0.01)	0.31
PFBA	0.03 ± 0.03	0.02 (0.01, 0.19)	0.04 ± 0.08	0.02 (0.01, 0.48)	0.92
PFHpA	0.03 ± 0.02	0.02 (0.01, 0.12)	0.03 ± 0.03	0.02 (0.01, 0.20)	0.46
PFOA	0.96 ± 0.49	0.96 (0.08, 2.27)	1.04 ± 0.52	1.03 (0.08, 2.33)	0.39
PFNA	0.35 ± 0.18	0.34 (0.03, 0.93)	0.41 ± 0.21	0.37 (0.04, 0.99)	0.12
PFDA	0.17 ± 0.09	0.16 (0.03, 0.47)	0.22 ± 0.11	0.20 (0.04, 0.58)	0.01
PFUdA	0.07 ± 0.04	0.06 (0.01, 0.18)	0.07 ± 0.04	0.06 (0.01, 0.20)	0.88
PFDoA	0.01 ± 0.01	0.01 (0.01, 0.08)	0.02 ± 0.03	0.01 (0.01, 0.26)	0.44
PFTrDA	0.01 ± 0.01	0.01 (0.01, 0.05)	0.01 ± 0.01	0.01 (0.01, 0.07)	0.63
PFTeDA	0.01 ± 0.00	0.01 (0.01, 0.03)	0.01 ± 0.02	0.01 (0.01, 0.12)	0.97
**Monohydroxylated PAH metabolites (µg/g creatinine)**					
1-OH-NAP	0.46 ± 0.56	0.27 (0.01, 2.90)	0.71 ± 1.33	0.35 (0.04, 9.04)	0.16
2-OH-NAP	5.43 ± 5.88	2.91 (1.01, 22.8)	3.83 ± 2.90	2.96 (0.77, 13.7)	0.49
2-OH-FLUO	0.39 ± 0.35	0.29 (0.01, 2.26)	0.29 ± 0.24	0.21 (0.11, 1.59)	0.02
1-OH-PHEN	0.23 ± 0.27	0.16 (0,06, 1.84)	0.20± 0.16	0.14 (0.04, 0.82)	0.52
2-OH-PHEN	0.09 ± 0.09	0.07 (0.02, 0.51)	0.13 ± 0.14	0.08 (0.03, 0.69)	0.02
3-OH-PHEN	0.12 ± 0.11	0.09 (0.01, 0.53)	0.09 ± 0.07	0.07 (0.02, 0.28)	0.12
4-OH-PHEN	0.03± 0.03	0.02 (0.00, 0.11)	0.03 ± 0.04	0.02 (0.00, 0.20)	0.46
9-OH-PHEN	0.09 ± 0.09	0.07 (0.00, 0.39)	0.10 ± 0.11	0.06 (0.02, 0.69)	0.78
1-OH–pyrene	0.07 ± 0.06	0.05 (0.01, 0.26)	0.07 ± 0.04	0.06 (0.01, 0.25)	0.42

Supplementary Table S3. Concentrations of plasma POPs and urinary PAH metabolites in samples collected in Ostrava, an industrial region

	Ostrava (N=54) (Season 1)		Ostrava (N=54) (Season 2)		
**Variable**	Mean ± SD	Median (min, max)	Mean ± SD	Median (min, max)	P
		**Polychlorinated biphenyls (ng/g lipid weight)**			
PCB 28	0.73 ± 0.99	0.05 (0.05, 4.02)	1.03 ± 1.41	0.65 (0.05, 6.16)	0.21
PCB 52	0.13 ± 0.13	0.05(0.05, 0.58)	0.17 ± 0.23	0.05 (0.05, 1.07)	0.61
PCB 101	0.36 ± 0.32	0.32 (0.05, 1.35)	0.14 ± 0.37	0.05 (0.05, 2.20)	<0.001
PCB 118	3.05 ± 2.38	2.41 (0.05, 13.55)	4.79 ± 3.19	4.20 (0.05, 17.50)	0.001
PCB 138	28.89 ± 26.31	21.15 (2.94, 159.2)	69.3 ± 53.0	60.8 (8.37, 280.0)	<0.001
PCB 153	71.1 ± 65.2	52.1 (5.76, 397.8)	128.5 ± 104.6	109.0 (14.8, 567.0)	<0.001
PCB 170	48.4 ± 45.5	33.2 (3.34, 281.1)	48.7 ± 39.3	40.1 (4.42, 227.0)	0.68
PCB 180	111.1 ± 104.3	77.3 (59.5, 652.9)	156.9 ± 135.0	122.0 (11.3, 717.0)	0.03
		**Organochlorinated pesticides (ng/g lipid weight)**			
o,p'-DDE	0.16 ± 0.18	0.05 (0.05, 1.07)	4.90 ± 35.3	0.05 (0.05, 257.3)	<0.001
p,p'-DDE	82.9 ± 87.4	62.0 (15.1, 587.1)	150.4 ± 134.6	117.00 (22.7, 873.0)	<0.001
o,p'-DDD	0.07 ± 0.11	0.05 (0.05, 0.81)	0.05 ± 0.00	0.05 (0.05, 0.05)	0.08
p,p'-DDD	0.72 ± 1.12	0.46 (0.05, 7.11)	2.09 ± 2.38	1.39 (0.05, 14.0)	<0.001
o,p'-DDT	0.25 ± 0.00	0.25 (0.25, 0.25)	0.35 ± 0.59	0.25 (0.25, 4.45)	0.15
p,p'-DDT	3.01 ± 6.94	1.14 (0.25, 49.7)	3.79 ± 14.6	0.25 (0.25, 101.0)	0.004
HCB	11.9 ± 6.42	11.1 (1.56, 33.1)	17.4 ± 10.1	14.6 (5.91, 52.5)	0.001
α-HCH	0.89 ± 1.00	0.55 (0.05, 4.51)	0.06 ± 0.07	0.05 (0.05, 0.57)	<0.001
β-HCH	2.92 ± 2.64	2.59 (0.05, 9.59)	5.74 ± 7.14	3.64 (0.05, 31.3)	0.21
γ-HCH	0.29 ± 0.42	0.05 (0.05, 1.89)	0.89 ± 4.44	0.05 (0.05, 29.2)	<0.001
		**Brominated flame retardants (ng/g lipid weight)**			
BDE 47	4.47 ± 4.37	2.82 (0.05, 15.0)	1.84 ± 1.85	1.51 (0.62, 14.1)	0.01
BDE 99	0.68 ± 3.44	0.15 (0.15, 25.4)	0.59 ± 1.22	0.15 (0.15, 8.11)	0.004
BDE 100	0.16 ± 0.06	0.15 (0.15, 0.54)	0.20 ± 0.27	0.15 (0.15, 2.00)	0.95
BDE 153	0.21 ± 0.21	0.15 (0.15, 1.07)	0.50 ± 0.67	0.15 (0.15, 3.24)	0.001
BDE 154	0.15 ± 0.00	0.15 (0.15, 0.15)	0.18 ± 0.19	0.15 (0.15, 1.55)	0.31

BDE 183	0.20 ± 0.25	0.15 (0.15, 1.62)	0.29 ± 0.50	0.15 (0.15, 2.67)	0.24
BDE 209	8.66 ± 34.4	1.33 (0.75, 251.0)	46.3 ± 90.6	0.75 (0.75, 354.0)	0.39
**Per- and polyfluoroalkylated substances (ng/mL plasma)**					
PFBS	0.005 ± 0.00	0.005 (0.005, 0.005)	0.006 ± 0.003	0.005 (0.005, 0.02)	0.02
PFHxS	0.57 ± 0.35	0.52 (0.05, 1.73)	0.58 ± 0.31	0.53 (0.04, 1.79)	0.73
PFOS	3.35 ± 3.20	2.46 (0.27, 16.6)	3.30 ± 3.45	2.25 (0.35, 18.4)	0.52
PFDS	0.005 ± 0.00	0.005 (0.005, 0.005)	0.005 ± 0.00	0.005 (0.005, 0.005)	1.00
PFBA	0.03 ± 0.04	0.02 (0.005, 0.33)	0.03 ± 0.02	0.03 (0.005, 0.17)	0.01
PFHpA	0.02 ± 0.02	0.02 (0.005, 0.08)	0.02 ± 0.02	0.01 (0.005, 0.15)	0.38
PFOA	0.98 ± 0.59	0.85 (0.10, 2.41)	0.98 ± 0.58	0.87 (0.11, 2.39)	0.91
PFNA	0.41 ± 0.35	0.34 (0.03, 2.07)	0.45 ± 0.45	0.36 (0.04, 3.00)	0.72
PFDA	0.15 ± 0.09	0.12 (0.03, 0.46)	0.16 ± 0.14	0.13 (0.03, 0.72)	0.89
PFUdA	0.07 ± 0.04	0.06 (0.02, 0.17)	0.07 ± 0.06	0.06 (0.02, 0.32)	0.54
PFDoA	0.01 ± 0.01	0.01 (0.005, 0.06)	0.02 ± 0.02	0.01 (0.005, 0.10)	0.77
PFTrDA	0.01 ± 0.01	0.005 (0.005, 0.06)	0.01 ± 0.01	0.005 (0.005, 0.09)	0.95
PFTeDA	0.005 ± 0.001	0.005 (0.005, 0.01)	0.006 ± 0.004	0.005 (0.005, 0.03)	0.16
**Monohydroxylated PAH metabolites (μg/g creatinine)**					
1-OH-NAP	0.54 ± 0.70	0.31 (0.008, 3.93)	1.45 ± 2.82	0.60 (0.13, 19.0)	0.001
2-OH-NAP	5.15 ± 4.93	3.99 (0.83, 27.0)	4.26 ± 3.09	3.41 (0.63, 18.0)	0.74
2-OH-FLUO	0.49 ± 0.39	0.37 (0.008, 1.66)	0.59 ± 0.89	0.30 (0.11, 5.91)	0.83
1-OH-PHEN	0.32 ± 0.25	0.23 (0.08, 1.14)	0.34 ± 0.29	0.23 (0.06, 1.65)	0.81
2-OH-PHEN	0.13 ± 0.11	0.08 (0.03, 0.60)	0.17 ± 0.14	0.13 (0.04, 0.78)	0.01
3-OH-PHEN	0.18 ± 0.16	0.15 (0.03, 0.90)	0.19 ± 0.19	0.12 (0.03, 1.13)	0.66
4-OH-PHEN	0.04 ± 0.03	0.03 (0.004, 0.17)	0.05 ± 0.04	0.03 (0.009, 0.25)	0.18
9-OH-PHEN	0.09 ± 0.07	0.06 (0.02, 0.31)	0.14 ± 0.16	0.08 (0.004, 0.72)	0.13
1-OH–pyrene	0.16 ± 0.16	0.10 (0.008, 0.84)	0.13 ± 0.09	0.11 (0.05, 0.41)	0.87

Supplementary Table S4. Associations of oxidative stress markers with environmental pollutants, parameters of antioxidant response and inflammation

	8-oxodG			15-F2t-IsoP		
	B, 95% CI	p-value	q-value	B, 95% CI	p-value	q-value
Age	0.007 (-0.01, 0.03)	0.49	0.83	-0.08 (-0.43, 0.27)	0.65	0.82
BMI	-0.03 (-0.07, 0.006)	0.11	0.37	-0.55 (-1.08, -0.03)	0.04	0.19
Cotinine	0.001 (0.000, 0.002)	0.01	0.12	-0.009 (-0.02, -0.001)	0.02	0.15
Education	-0.16 (-0.65, 0.33)	0.52	0.83	-1.18 (-7.29, 4.93)	0.71	0.87
Locality	0.02 (-0.22, 0.27)	0.87	0.92	5.07 (1.76, 8.38)	0.003	0.04
B[a]P	-0.09 (-0.29, 0.12)	0.40	0.78	-3.99 (-9.00, 1.01)	0.12	0.39
PM2.5	0.02 (0.004, 0.04)	0.01	0.12	-0.18 (-0.39, 0.03)	0.09	0.33
Ozone	0.004 (-0.002, 0.01)	0.24	0.61	-0.007 (-0.087, 0.073)	0.86	0.96
SOD	0.00 (-0.01, 0.01)	0.94	0.96	0.05 (-0.08, 0.18)	0.48	0.77
CAT	-0.001 (-0.01, 0.009)	0.80	0.92	0.20 (0.05, 0.34)	0.007	0.06
GPX	0.002 (-0.001, 0.005)	0.14	0.40	-0.02 (-0.06, 0.01)	0.20	0.49
ORAC	-0.20 (-0.41, 0.01)	0.06	0.24	-0.75 (-3.43, 1.94)	0.59	0.78
TNF-α	0.001 (-0.002, 0.003)	0.53	0.83	-0.002 (-0.05, 0.04)	0.91	0.96
IL-1β	0.000 (-0.002, 0.001)	0.89	0.92	0.02 (-0.001, 0.04)	0.07	0.27
IL-6	0.002 (0.001, 0.003)	<0.001	<0.001	0.04 (0.03, 0.05)	<0.001	<0.001
PCB 28	-0.07 (-0.15, 0.02)	0.11	0.37	-0.69 (-2.28, 0.90)	0.40	0.68
PCB 52	0.40 (-0.26, 1.06)	0.24	0.63	-2.33 (-7.60, 2.97)	0.39	0.68
PCB 101	0.03 (-0.20, 0.26)	0.78	0.92	-4.94 (-8.60, -1.28)	0.008	0.06
PCB 118	0.006 (-0.06, 0.07)	0.85	0.92	-0.02 (-0.82, 0.78)	0.96	0.96
PCB 180	0.000 (-0.002, 0.001)	0.77	0.92	0.007 (-0.02, 0.03)	0.59	0.78
o,p'-DDE	-0.004 (-0.005, -0.003)	<0.001	<0.001	-0.01 (-0.02, -0.001)	0.04	0.19

p,p'-DDE	0.000 (-0.001, 0.002)	0.49	0.83	-0.005 (-0.02, 0.01)	0.59	0.78
o,p'-DDD	1.49 (0.25, 2.72)	0.02	0.14	10.02 (-13.31, 33.36)	0.40	0.68
p,p'-DDD	-0.01 (-0.05, 0.03)	0.58	0.84	0.46 (-0.06, 0.98)	0.08	0.31
o,p'-DDT	0.07 (-0.27, 0.41)	0.70	0.92	0.36 (-5.27, 5.99)	0.90	0.96
p,p'-DDT	-0.003 (-0.01, 0.005)	0.41	0.78	0.05 (-0.06, 0.16)	0.39	0.68
HCB	0.009 (-0.009, 0.03)	0.32	0.77	-0.08 (-0.35, 0.19)	0.55	0.78
α-HCH	-0.12 (-0.33, 0.09)	0.26	0.65	3.89 (-1.18, 8.96)	0.13	0.40
β-HCH	0.02 (-0.02, 0.05)	0.36	0.78	-0.20 (-0.60, 0.19)	0.31	0.62
γ-HCH	0.01 (-0.01, 0.03)	0.41	0.78	-0.25 (-0.58, 0.09)	0.14	0.40
BDE 47	0.004 (-0.04, 0.05)	0.85	0.92	-0.73 (-1.23, -0.22)	0.005	0.05
BDE 99	0.02 (-0.04, 0.07)	0.52	0.83	3.42 (1.97, 4.88)	<0.001	<0.001
BDE 100	-0.73 (-1.36, -0.09)	0.03	0.15	-5.39 (-10.44, -0.34)	0.04	0.19
BDE 153	0.04 (0.009, 0.07)	0.01	0.12	-0.21 (-0.82, 0.40)	0.50	0.78
BDE 154	-1.00 (-1.47, -0.54)	<0.001	<0.001	-8.15 (-10.60, -5.71)	<0.001	<0.001
BDE 209	0.001 (-0.002, 0.004)	0.67	0.92	-0.01 (-0.02, 0.004)	0.18	0.47
PFBS	-4.88 (-16.24, 6.49)	0.40	0.78	-91.8 (-183.8, 0.35)	0.05	0.23
PFHxS	0.60 (0.04, 1.16)	0.04	0.17	0.25 (-6.69, 7.19)	0.94	0.96
PFOS	0.05 (0.003, 0.11)	0.04	0.17	-0.48 (-1.34, 0.39)	0.28	0.61
PFDS	-9.02 (-74.16, 56.12)	0.79	0.92	270.6 (-836.2, 1377.4)	0.63	0.82
PFBA	4.62 (0.63, 8.61)	0.02	0.15	-3.83 (-57.0, 49.3)	0.89	0.96
PFHpA	5.51 (-1.60, 12.62)	0.13	0.40	-44.7 (-118.3, 28.9)	0.23	0.54
PFOA	0.30 (-0.01, 0.61)	0.06	0.24	-3.29 (-7.61, 1.04)	0.14	0.40
PFDA	-0.10 (-1.39, 1.18)	0.88	0.92	-6.72 (-23.57, 10.13)	0.43	0.72
PFUdA	-0.63 (-4.20, 2.94)	0.73	0.92	-25.1 (-72.9, 22.8)	0.31	0.62
1-OH-NAP	-0.06 (-0.12, -0.003)	0.04	0.17	-0.06 (-0.89, 0.77)	0.89	0.96
2-OH-NAP	-0.02 (-0.05, 0.01)	0.23	0.63	0.04 (-0.36, 0.44)	0.83	0.96
2-OH-FLUO	0.07 (-0.19, 0.33)	0.59	0.84	-0.48 (-4.38, 3.43)	0.81	0.96
1-OH-PHEN	0.12 (-0.48, 0.71)	0.71	0.92	0.97 (-7.62, 9.56)	0.83	0.96

2-OH-PHEN	0.005 (-1.30, 1.30)	1.00	1.00	-5.03 (-21.35, 11.29)	0.55	0.78
3-OH-PHEN	-0.30 (-1.19, 0.60)	0.52	0.83	9.44 (-4.59, 23.46)	0.19	0.47
4-OH-PHEN	-1.48 (-4.50, 1.54)	0.34	0.78	1.21 (-42.65, 45.08)	0.96	0.96
9-OH-PHEN	0.36 (-0.89, 1.62)	0.57	0.84	-7.66 (-21.79, 6.46)	0.29	0.61
1-OH–pyrene	0.24 (-1.84, 2.31)	0.82	0.92	6.72 (-17.66, 31.10)	0.59	0.78
